# Diaqua­bis(5-carb­oxy-1*H*-pyrazole-3-carboxyl­ato-κ^2^ *N* ^2^,*O* ^3^)cobalt(II) dihydrate

**DOI:** 10.1107/S1600536809029456

**Published:** 2009-07-31

**Authors:** Hui-Dong Xie, Li Jin, Cheng-Zhi Xie

**Affiliations:** a School of Science, Xi’an University of Architecture & Technology, Xi’an 710055, People’s Republic of China; b Tianjin Medical University, Tianjin 300070, People’s Republic of China

## Abstract

In the title complex, [Co(C_5_H_3_N_2_O_4_)_2_(H_2_O)_2_]·2H_2_O, the Co^II^ ion lies on an inversion center and is coordinated in a distorted octa­hedral environment. In the crystal structure, complex and water mol­ecules are linked into a three-dimensional network by O—H⋯O and N—H⋯O hydrogen bonds.

## Related literature

For a mononuclear zinc(II) complex with a pyrazole-3,5-dicarboxyl­ato ligand, see: Xie *et al.* (2006 ▶) and for a cobalt(III) complex with a 5-carb­oxy-1*H*-pyrazole-3-carboxyl­ato ligand, see: Xie *et al.* (2007 ▶). The 3,5-pyrazole­dicarboxylic acid ligand is asymmetric and has six potential coordination sites which can act to link together metal centers through a number of bridging modes, see: King *et al.* (2004 ▶). A variety of complexes containing this ligand have been reported, see: Frisch & Cahill (2005 ▶); King *et al.* (2003 ▶, 2004 ▶); Li *et al.* (2005 ▶); Pan, Ching *et al.* (2001 ▶); Pan, Frydel *et al.* (2001 ▶).
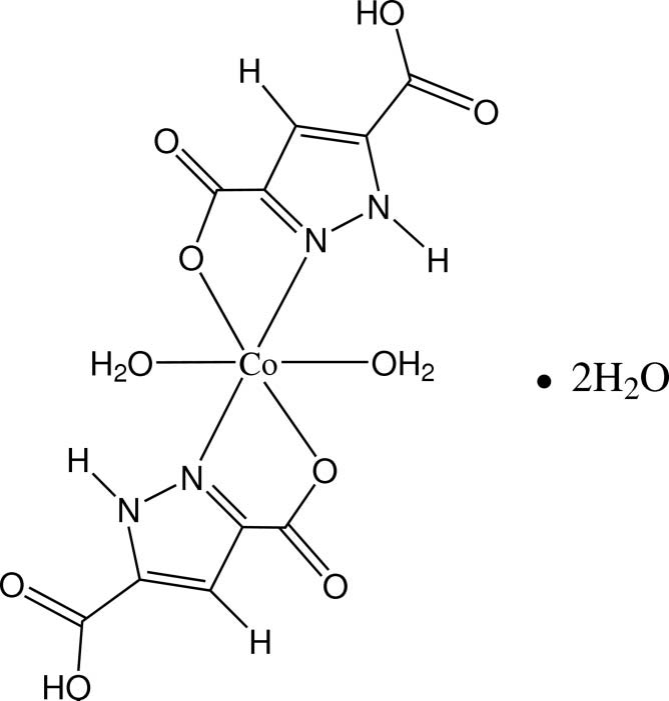


## Experimental

### 

#### Crystal data

[Co(C_5_H_3_N_2_O_4_)_2_(H_2_O)_2_]·2H_2_O
*M* *_r_* = 441.18
Monoclinic, 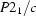

*a* = 10.030 (3) Å
*b* = 12.483 (4) Å
*c* = 6.827 (2) Å
β = 108.641 (4)°
*V* = 809.9 (5) Å^3^
*Z* = 2
Mo *K*α radiation
μ = 1.14 mm^−1^
*T* = 291 K
0.32 × 0.27 × 0.14 mm

#### Data collection

Bruker SMART CCD diffractometer
Absorption correction: multi-scan (*SADABS*; Sheldrick, 1996 ▶) *T*_min_ = 0.713, *T*_max_ = 0.854
5748 measured reflections
1502 independent reflections
1331 reflections with *I* > 2σ(*I*)
*R*_int_ = 0.036

#### Refinement

*R*[*F* ^2^ > 2σ(*F* ^2^)] = 0.048
*wR*(*F* ^2^) = 0.142
*S* = 1.12
1502 reflections
129 parameters
H atoms treated by a mixture of independent and constrained refinement
Δρ_max_ = 0.84 e Å^−3^
Δρ_min_ = −0.44 e Å^−3^

### 

Data collection: *SMART* (Bruker, 2001 ▶); cell refinement: *SAINT* (Bruker, 2001 ▶); data reduction: *SAINT*; program(s) used to solve structure: *SHELXS97* (Sheldrick, 2008 ▶); program(s) used to refine structure: *SHELXL97* (Sheldrick, 2008 ▶); molecular graphics: *SHELXTL* (Sheldrick, 2008 ▶); software used to prepare material for publication: *SHELXTL*.

## Supplementary Material

Crystal structure: contains datablocks I, global. DOI: 10.1107/S1600536809029456/lh2869sup1.cif

Structure factors: contains datablocks I. DOI: 10.1107/S1600536809029456/lh2869Isup2.hkl

Additional supplementary materials:  crystallographic information; 3D view; checkCIF report

## Figures and Tables

**Table:** 

Co1—O5	2.065 (3)
Co1—N1	2.108 (3)
Co1—O1	2.120 (3)
O1—C1	1.262 (5)
O2—C1	1.256 (5)

**Table:** 

O5^i^—Co1—O5	180
O5—Co1—N1	90.84 (12)
O5—Co1—N1^i^	89.16 (12)
N1—Co1—N1^i^	180
O5—Co1—O1^i^	91.18 (12)
N1—Co1—O1^i^	103.22 (11)
O5—Co1—O1	88.82 (12)
N1—Co1—O1	76.78 (11)
O1^i^—Co1—O1	180

Symmetry code: (i) 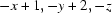.

**Table 2:** Hydrogen-bond geometry (Å, °)

*D*—H⋯*A*	*D*—H	H⋯*A*	*D*⋯*A*	*D*—H⋯*A*
O4—H4⋯O2^ii^	0.82	1.73	2.535 (4)	169
O5—H1*W*⋯O3^iii^	0.83	2.07	2.887 (4)	171
O5—H2*W*⋯O2^iv^	0.83	1.91	2.726 (4)	171
O6—H4*W*⋯O1^v^	0.85 (11)	2.06 (11)	2.828 (5)	149 (10)
O6—H3*W*⋯O3^vi^	0.84	2.30	2.932 (5)	132
N2—H2⋯O6^vii^	0.86	1.91	2.714 (5)	155

Symmetry codes: (ii) 
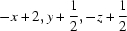
; (iii) 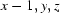; (iv) 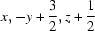; (v) 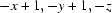; (vi) 
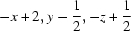
; (vii) 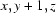.

**Fig. 1.**
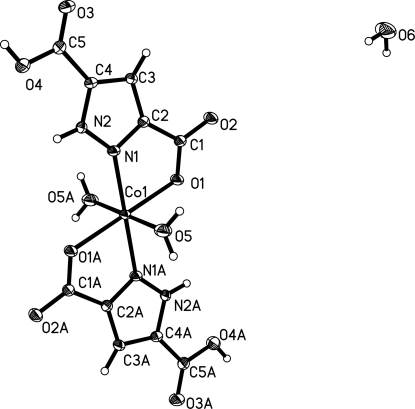
The molecular structure of (I), with atom labels and 35% probability displacement ellipsoids for non-H atoms [symmetry code: (A) -x+1, -y+2, -z]. Only the unique solvent water molecule is shown.

**Fig. 2.**
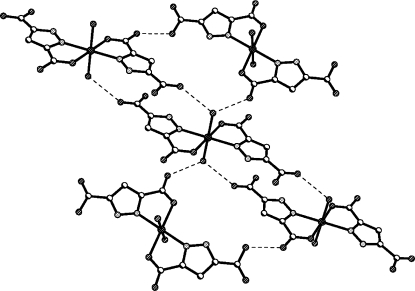
Part of the crystal structure of (I) showing the donor acceptor distances of hydrogen bonds as dashed lines. H atoms have been omitted for clarity.

## supplementary crystallographic information

### Comment

In the past few decades, self-assembly processes involving metal ions and organic ligands directed by either metal coordination or hydrogen bonds have received a great deal of attention in the field of supramolecular chemistry and crystal engineering. The 3,5-pyrazoledicarboxylic acid ligand is asymmetric and has six potential coordination sites which can act to link together metal centers through a number of bridging modes (King *et al.*, 2004). A variety of complexes containing this ligand have been reported (Frisch *et al.*, 2005; King *et al.*, 2003, 2004; Pan, Ching *et al.*, 2001; Pan, Frydel *et al.*, 2001; Li *et al.*, 2005).

The molecular structure of the title complex , (I), is shown in Fig. 1. The Co^II^ ion is located on an inversion center and is coordinated in a distorted octahedral environment. The axial sites are occupied by water molecules and the equatorial plane is fromed by two oxygen donors and two nitrogen donors from two chelating 5-carboxy-pyrazole-3-carboxylato ligands. In the crystal structure complex and water molecules are linked into a three-dimensional network by O-H···O and N-H···O hydrogen bonds.

### Experimental

A mixture of cobalt(II) nitrate (hexhydrate) (0.2 mmol, 58 mg), 3,5-pyrazoledicarboxylic acid (0.4 mmol, 62 mg) and  H_2_O (18.0 ml) in a 1:2:5000 molar ratio was sealed in a 25 ml stainless steel reactor with a Teflon  liner. The autoclave was kept at 423 K for 3 d, then cooled to room temperature at a rate of 4 K/h. Orange block-shaped crystals of the title complex were collected by filtration for the structural analysis.

### Refinement

All H atoms bonded to C and N atoms were initially located in difference Fourier maps but were subsequently refined in a riding-model approximation with C—H = 0.93 Å, N—H = 0.86 Å, *U*_iso_(H) = 1.2*U*_eq_(C,N). The O atoms bonded to the carboxylic group and the coordinated water atom were included in calculated positions and refined in a riding-model approximation with O-H = 0.82-0.83Å and *U*_iso_(H) = 1.2-1.5*U*_eq_(O). One of the solvent water H atoms was included with O-H = 0.84; *U*_iso_(H) = 1.2*U*_eq_(O) and the other H atom was refined isotropically.

### Crystal data

**Table:**

[Co(C_5_H_3_N_2_O_4_)_2_(H_2_O)_2_]·2H_2_O	*F*(000) = 450
*M**_r_* = 441.18	*D*_x_ = 1.809 Mg m^−^^3^
Monoclinic, *P*2_1_/*c*	Mo *K*α radiation, λ = 0.71073 Å
Hall symbol: -P 2ybc	Cell parameters from 2747 reflections
*a* = 10.030 (3) Å	θ = 2.7–27.9°
*b* = 12.483 (4) Å	µ = 1.14 mm^−^^1^
*c* = 6.827 (2) Å	*T* = 291 K
β = 108.641 (4)°	Block, orange
*V* = 809.9 (5) Å^3^	0.32 × 0.27 × 0.14 mm
*Z* = 2	

### Data collection

**Table:**

Bruker SMART CCD diffractometer	1502 independent reflections
Radiation source: fine-focus sealed tube	1331 reflections with *I* > 2σ(*I*)
graphite	*R*_int_ = 0.036
φ and ω scans	θ_max_ = 25.5°, θ_min_ = 2.7°
Absorption correction: multi-scan (*SADABS*; Sheldrick, 1996)	*h* = −12→12
*T*_min_ = 0.713, *T*_max_ = 0.854	*k* = −15→15
5748 measured reflections	*l* = −8→8

### Refinement

**Table:**

Refinement on *F*^2^	Primary atom site location: structure-invariant direct methods
Least-squares matrix: full	Secondary atom site location: difference Fourier map
*R*[*F*^2^ > 2σ(*F*^2^)] = 0.048	Hydrogen site location: inferred from neighbouring sites
*wR*(*F*^2^) = 0.142	H atoms treated by a mixture of independent and constrained refinement
*S* = 1.12	*w* = 1/[σ^2^(*F*_o_^2^) + (0.0775*P*)^2^ + 1.4115*P*] where *P* = (*F*_o_^2^ + 2*F*_c_^2^)/3
1502 reflections	(Δ/σ)_max_ < 0.001
129 parameters	Δρ_max_ = 0.84 e Å^−^^3^
0 restraints	Δρ_min_ = −0.44 e Å^−^^3^

### Special details

**Table:**

Geometry. All e.s.d.'s (except the e.s.d. in the dihedral angle between two l.s. planes) are estimated using the full covariance matrix. The cell e.s.d.'s are taken into account individually in the estimation of e.s.d.'s in distances, angles and torsion angles; correlations between e.s.d.'s in cell parameters are only used when they are defined by crystal symmetry. An approximate (isotropic) treatment of cell e.s.d.'s is used for estimating e.s.d.'s involving l.s. planes.
Refinement. Refinement of *F*^2^ against ALL reflections. The weighted *R*-factor *wR* and goodness of fit *S* are based on *F*^2^, conventional *R*-factors *R* are based on *F*, with *F* set to zero for negative *F*^2^. The threshold expression of *F*^2^ > σ(*F*^2^) is used only for calculating *R*-factors(gt) *etc*. and is not relevant to the choice of reflections for refinement. *R*-factors based on *F*^2^ are statistically about twice as large as those based on *F*, and *R*- factors based on ALL data will be even larger.

### Fractional atomic coordinates and isotropic or equivalent isotropic displacement parameters (Å^2^)

**Table:**

	*x*	*y*	*z*	*U*_iso_*/*U*_eq_	
Co1	0.5000	1.0000	0.0000	0.0227 (3)	
O1	0.5183 (3)	0.8358 (2)	−0.0667 (5)	0.0308 (6)	
O2	0.6657 (3)	0.6977 (2)	−0.0142 (5)	0.0337 (7)	
O3	1.1998 (3)	0.9569 (3)	0.3134 (5)	0.0408 (8)	
O4	1.0960 (3)	1.1105 (2)	0.3529 (5)	0.0366 (7)	
H4	1.1772	1.1324	0.3977	0.055*	
O5	0.4797 (3)	0.9554 (3)	0.2803 (5)	0.0378 (7)	
H1W	0.4031	0.9594	0.3020	0.045*	
H2W	0.5296	0.9069	0.3484	0.045*	
O6	0.7621 (4)	0.2301 (3)	0.2670 (8)	0.0684 (13)	
H3W	0.7617	0.2926	0.3116	0.082*	
N1	0.7187 (3)	0.9727 (3)	0.1137 (5)	0.0238 (7)	
N2	0.8371 (3)	1.0290 (2)	0.1925 (5)	0.0237 (7)	
H2	0.8402	1.0958	0.2251	0.028*	
C1	0.6402 (4)	0.7957 (3)	−0.0049 (6)	0.0239 (8)	
C2	0.7586 (4)	0.8723 (3)	0.0850 (6)	0.0232 (7)	
C3	0.9054 (4)	0.8646 (3)	0.1462 (6)	0.0255 (8)	
H3	0.9595	0.8047	0.1422	0.031*	
C4	0.9519 (4)	0.9668 (3)	0.2143 (6)	0.0246 (8)	
C5	1.0964 (4)	1.0103 (3)	0.2981 (6)	0.0264 (8)	
H4W	0.678 (12)	0.232 (9)	0.182 (18)	0.19 (4)*	

### Atomic displacement parameters (Å^2^)

**Table:**

	*U*^11^	*U*^22^	*U*^33^	*U*^12^	*U*^13^	*U*^23^
Co1	0.0135 (4)	0.0169 (4)	0.0356 (4)	0.0024 (2)	0.0049 (3)	0.0001 (3)
O1	0.0156 (13)	0.0203 (13)	0.0522 (17)	0.0011 (10)	0.0046 (11)	−0.0035 (12)
O2	0.0203 (14)	0.0210 (14)	0.0535 (18)	0.0006 (10)	0.0031 (12)	−0.0062 (12)
O3	0.0198 (15)	0.0342 (16)	0.066 (2)	0.0006 (12)	0.0106 (14)	−0.0041 (15)
O4	0.0207 (14)	0.0269 (15)	0.0568 (19)	−0.0042 (11)	0.0048 (13)	−0.0063 (13)
O5	0.0292 (16)	0.0418 (17)	0.0443 (16)	0.0129 (13)	0.0143 (13)	0.0130 (14)
O6	0.041 (2)	0.0318 (19)	0.124 (4)	0.0026 (15)	0.014 (2)	−0.010 (2)
N1	0.0136 (15)	0.0210 (15)	0.0344 (17)	0.0010 (12)	0.0041 (12)	−0.0024 (12)
N2	0.0159 (15)	0.0154 (14)	0.0379 (17)	−0.0022 (12)	0.0059 (13)	−0.0025 (13)
C1	0.0170 (17)	0.0197 (18)	0.0327 (19)	−0.0005 (14)	0.0047 (15)	−0.0014 (14)
C2	0.0167 (17)	0.0185 (17)	0.0329 (19)	−0.0012 (14)	0.0056 (15)	−0.0014 (14)
C3	0.0171 (17)	0.0183 (18)	0.039 (2)	0.0018 (13)	0.0066 (15)	−0.0005 (15)
C4	0.0154 (17)	0.0236 (18)	0.0336 (19)	0.0010 (14)	0.0064 (14)	0.0008 (15)
C5	0.0205 (19)	0.0238 (19)	0.033 (2)	−0.0021 (14)	0.0065 (16)	0.0010 (15)

### Geometric parameters (Å, °)

**Table:**

Co1—O5^i^	2.065 (3)	O5—H2W	0.8277
Co1—O5	2.065 (3)	O6—H3W	0.8380
Co1—N1	2.108 (3)	O6—H4W	0.85 (11)
Co1—N1^i^	2.108 (3)	N1—N2	1.336 (4)
Co1—O1^i^	2.120 (3)	N1—C2	1.349 (5)
Co1—O1	2.120 (3)	N2—C4	1.358 (5)
O1—C1	1.262 (5)	N2—H2	0.8600
O2—C1	1.256 (5)	C1—C2	1.495 (5)
O3—C5	1.209 (5)	C2—C3	1.400 (5)
O4—C5	1.306 (5)	C3—C4	1.386 (5)
O4—H4	0.8200	C3—H3	0.9300
O5—H1W	0.8288	C4—C5	1.481 (5)
			
O5^i^—Co1—O5	180	N2—N1—C2	106.3 (3)
O5^i^—Co1—N1	89.16 (12)	N2—N1—Co1	138.6 (3)
O5—Co1—N1	90.84 (12)	C2—N1—Co1	114.8 (2)
O5^i^—Co1—N1^i^	90.84 (12)	N1—N2—C4	110.9 (3)
O5—Co1—N1^i^	89.16 (12)	N1—N2—H2	124.6
N1—Co1—N1^i^	180	C4—N2—H2	124.6
O5^i^—Co1—O1^i^	88.82 (12)	O2—C1—O1	124.1 (3)
O5—Co1—O1^i^	91.18 (12)	O2—C1—C2	119.7 (3)
N1—Co1—O1^i^	103.22 (11)	O1—C1—C2	116.2 (3)
N1^i^—Co1—O1^i^	76.78 (11)	N1—C2—C3	110.7 (3)
O5^i^—Co1—O1	91.18 (12)	N1—C2—C1	114.8 (3)
O5—Co1—O1	88.82 (12)	C3—C2—C1	134.5 (3)
N1—Co1—O1	76.78 (11)	C4—C3—C2	104.3 (3)
N1^i^—Co1—O1	103.22 (11)	C4—C3—H3	127.9
O1^i^—Co1—O1	180	C2—C3—H3	127.9
C1—O1—Co1	116.9 (2)	N2—C4—C3	107.9 (3)
C5—O4—H4	109.5	N2—C4—C5	121.6 (3)
Co1—O5—H1W	121.4	C3—C4—C5	130.6 (3)
Co1—O5—H2W	119.8	O3—C5—O4	125.8 (4)
H1W—O5—H2W	111.9	O3—C5—C4	122.5 (3)
H3W—O6—H4W	95.7	O4—C5—C4	111.7 (3)
			
O5^i^—Co1—O1—C1	92.3 (3)	Co1—N1—C2—C3	174.1 (3)
O5—Co1—O1—C1	−87.7 (3)	N2—N1—C2—C1	−179.5 (3)
N1—Co1—O1—C1	3.4 (3)	Co1—N1—C2—C1	−5.3 (4)
N1^i^—Co1—O1—C1	−176.6 (3)	O2—C1—C2—N1	−171.3 (4)
O5^i^—Co1—N1—N2	81.6 (4)	O1—C1—C2—N1	8.3 (5)
O5—Co1—N1—N2	−98.4 (4)	O2—C1—C2—C3	9.5 (7)
O1^i^—Co1—N1—N2	−6.9 (4)	O1—C1—C2—C3	−170.8 (4)
O1—Co1—N1—N2	173.1 (4)	N1—C2—C3—C4	0.1 (4)
O5^i^—Co1—N1—C2	−90.0 (3)	C1—C2—C3—C4	179.3 (4)
O5—Co1—N1—C2	90.0 (3)	N1—N2—C4—C3	−0.1 (4)
O1^i^—Co1—N1—C2	−178.6 (3)	N1—N2—C4—C5	179.8 (3)
O1—Co1—N1—C2	1.4 (3)	C2—C3—C4—N2	0.0 (4)
C2—N1—N2—C4	0.2 (4)	C2—C3—C4—C5	−179.9 (4)
Co1—N1—N2—C4	−171.9 (3)	N2—C4—C5—O3	−178.0 (4)
Co1—O1—C1—O2	172.5 (3)	C3—C4—C5—O3	1.9 (7)
Co1—O1—C1—C2	−7.2 (4)	N2—C4—C5—O4	2.7 (5)
N2—N1—C2—C3	−0.2 (4)	C3—C4—C5—O4	−177.4 (4)

Symmetry codes: (i) −*x*+1, −*y*+2, −*z*.

### Hydrogen-bond geometry (Å, °)

**Table:**

*D*—H···*A*	*D*—H	H···*A*	*D*···*A*	*D*—H···*A*
O4—H4···O2^ii^	0.82	1.73	2.535 (4)	169
O5—H1W···O3^iii^	0.83	2.07	2.887 (4)	171
O5—H2W···O2^iv^	0.83	1.91	2.726 (4)	171
O6—H4W···O1^v^	0.85 (11)	2.06 (11)	2.828 (5)	149 (10)
O6—H3W···O3^vi^	0.84	2.30	2.932 (5)	132
N2—H2···O6^vii^	0.86	1.91	2.714 (5)	155

Symmetry codes: (ii) −*x*+2, *y*+1/2, −*z*+1/2; (iii) *x*−1, *y*, *z*; (iv) *x*, −*y*+3/2, *z*+1/2; (v) −*x*+1, −*y*+1, −*z*; (vi) −*x*+2, *y*−1/2, −*z*+1/2; (vii) *x*, *y*+1, *z*.
